# Meeting report: British Society for Research on Ageing (BSRA) annual scientific meeting 2012, Aston University, Birmingham, 3rd to 4th July 2012

**DOI:** 10.1186/2046-2395-2-6

**Published:** 2013-04-05

**Authors:** Hannah Greenwood, David B Bartlett

**Affiliations:** 1 MRC-ARUK Centre for Musculoskeletal Ageing Research, School of Immunity and Infection, University of Birmingham, Birmingham, B15 2TT, UK

## Abstract

The focus of the British Society for Research on Ageing (BSRA) annual scientific meeting 2012 was aging mechanisms and mitigants. The themes covered included epigenetics, stem cells and regeneration, aging pathways and molecules, the aging bladder and bowel, as well as updates from the New Dynamics of Ageing (NDA) programme. The topics incorporated new directions for staple aging research in caloric restriction (CR), inflammation, immunesenescence, neurodegeneration, homeostasis and stress resistance, as well as newer research areas such as bioengineering of tissues, including the internal anal sphincter and thymus.

## Introduction

The main focus of the meeting was aging mechanisms and mitigants. The programme covered a number of topics including epigenetics of aging, the aging bowel and bladder, immunesenescence, and considered pathways and molecules involved in the aging process. This meeting report aims to provide a general overview of the topics discussed and the reader is referred to the cited references to gain further insight into the meeting’s content.

### Epigenetics and lifestyle influences on aging

Epidemiological studies from many of the well-established longitudinal cohorts have indicated that 25% of an individual’s longevity is influenced by genetics, and the remainder by environmental and lifestyle factors. This session focused on epigenetics and its contribution to understanding the mechanisms underlying lifestyle influences on longevity. Epigenetics has many definitions; however, the concept of genes and environmental stressors causing adaptations, which determine our susceptibility to disease, is perhaps most fitting when discussing aging and epigenetics.

The keynote address was given by Professor Osborne Almeida (Max Planck Institute of Psychiatry, Munich, Germany). Professor Almeida discussed the gene-environment interactions which define and influence brain and somatic aging, and in particular the role of stress and metabolic modifiers. He introduced the concept that genes account for large variations in human lifespan and that it is as likely that extended lifespan is due to the absence of harmful genes as the presence of life-extending genes. In 1936, Hans Selye suggested that stress is a major cause of disease and adaptation, and that our ability to deal with the body’s responses to stress defines the outcome of stress exposure. Professor Almeida proposed the physiological response, in the form of hyper-production of glucocorticoids (GC) via activation of the hypothalamic-pituitary-adrenal (HPA) axis, caused a number of cognitive impairments, for example reduced memory in response to GC [1] which is due to GC-induced damage. Indeed, stress-induced GC production induces pathology similar to Alzheimer’s disease in the form of amyloid-β deposition [2]. His data also suggests a link between stress and GC induction of depression and dementia, which is cumulative with each stressful event and therefore likely to have an epigenetic basis. Memory has been shown to be linked with DNA coding in neurons, and histone and DNA methylation may mark DNA to affect memory [3]. In young rodent models of stress he showed that increased GC production, depression and reduced learning were associated with reduced DNA methylation at the arginine vasopressin (AVP) locus, which causes increased upregulation of AVP expression in the parvocellular subdivision of the hypothalamic paraventricular nucleus and chronic hyperactivity of the HPA axis [4]. Therefore, early life stressors increase GC production via reduced methylation at the AVP locus, which can ultimately increase responses to stress in later life.

Professor Nektarios Tavernarakis (Institute of Molecular Biology and Biotechnology, Heraklion, Greece) followed on with a report on autophagy (cellular removal of damaged proteins and organelles) and some of the life course modulators of autophagy. Since the aging process is a consequence of the accumulation of molecular damage, increasing the risk of frailty and disease, autophagy is considered to play a role by removing damaged elements to regain homeostasis. Professor Tavernarakis used caloric restriction (CR) to investigate the role of autophagy in longevity, and showed that both CR and reduced insulin signaling increased *Caenorhabditis elegans* lifespan approximately two-fold. Combining the two resulted in a six to seven-fold increase (equal to around 500 years in humans). Reduced insulin/insulin-like growth factor (IGF) signaling during CR led to increased autophagy and longevity [5]. CR induces a decrease in mammalian target of rapamycin (mTOR) signaling and an increase in sirtuin activation. If autophagy is inhibited during CR then sirtuin activation is reduced, supporting the role of sirtuins in autophagy and CR-induced longevity [6].

Dr Werner Zwerschke (Cell Metabolism and Differentiation Research Group, Austrian Academy of Sciences, Innsbruck, Austria) developed these concepts further by discussing the signaling crosstalk involved with CR-induced longevity. He highlighted the cellular and biochemical processes altered by reduced insulin/IGF and mTOR signaling during CR, which are associated with longevity, and these include: reduced protein translation, increased detoxification pathways, increased autophagy and increased resistance to oxidative stress [7]. Furthermore, Dr Zwerschke described how mutations, which can reduce insulin/IGF and mTOR signaling, can influence morbidity and mortality in humans, particularly by lowering pleiotropic actions on cells, and reducing risk of developing cancers and cardiovascular disease [8]. Polymorphisms in IGF-1 receptor and FOXO3A are associated with increased lifespan and are more abundant in centenarians, which may infer longevity through an enhanced oxidative stress mechanism [9,10]. Importantly, these mutations and polymorphisms may be further compounded by environmental conditions in early life, since IGF-1 and growth hormone (GH) levels during early life are associated with increased lifespan [11]. A key factor which may play a role in mediating this effect is the accumulation of adipose tissue [12]. In particular, white adipose tissue located viscerally around the major organs is associated with increased insulin/IGF signaling and reduced lifespan. Partial mechanisms underlying these effects are revealed by fat-specific insulin receptor knockout (FIRKO) mice, which are protected from age-related loss of insulin sensitivity, diabetes and obesity, and show altered production of the adipokines adiponectin and leptin [13]. Adiponectin is negatively correlated whilst leptin is positively correlated with body fat and also associated with increased lifespan. Signaling through the insulin/IGF axis is associated with longevity and it is becoming clear that lifestyle can affect these processes from an early age.

This session was concluded by Dr Gro Amdam (School of Life Sciences, Arizona State University, Tempe, AZ, USA) who presented data regarding the use of the honey bee as a model of social interactions and their effects on aging [14]. In the beehive there is a distinct social hierarchy, with clear roles and progression of role adoption with age. All bees start as nurses, taking care of the offspring and habitat before some progress to foragers responsible for food collection. Interestingly, the lifespan of nurses is around 50 days whilst the lifespan of foragers is around 7 days. Dr Amdam’s work suggests this difference is due to social resource transfers among bees, which implies a social environment effect on aging. Nurse bees have reduced levels of lipofuscin, a marker of oxidative damage, as well as increased learning capacity over forager bees. However, Dr Amdam showed that if the social environment is altered by reducing the number of nurses, then the foragers revert back to being nurses and with this reverse their learning deficits are overcome [15]. This plasticity is considered to be mediated by a doubling of peroxiredoxins in the brain upon role reversal in bees, which then slows neuronal death.

### Understanding how cellular aging impacts on function in complex tissues: aging bowel and bladder

This session was a series of talks from researchers funded by the Biotechnology and Biological Sciences Research Council (BBSRC) initiative. The discussion aimed to try and improve the understanding of the basis of age-related decline in bladder and bowel function, and loss of continence. Dr Tamas Ordog (Center for Individualized Medicine, Mayo Clinic, Rochester, MN, USA) opened the session by discussing the impact of aging and CR on the differentiation and function of interstitial cells of Cajal (ICC) in the gut. ICC are mesodermal electrical pacemakers and neuromodulators responsible for regulating basal rhythm leading to contraction. Loss of ICC results in gastric neuromuscular dysfunction and ultimately reduced nutrient intake via early satiation [16]. Furthermore, there is a linear age-related ICC decline equating to around 1% per year [17]. The Klotho knockout mouse manifests a premature aging phenotype and was shown to have reduced food intake, reduced neurotransmission and reduced gastric compliance but not emptying. Dr Ordog’s group has shown that the Klotho mouse has reduced numbers of ICC suggesting an age-dependent loss of ICC [18]. In protein-energy malnutrition there is also a reduced number of ICC, further augmenting ICC association with nutrient-sensing mechanisms. These data suggest that aging associated with gastric dysfunction mimics CR in reducing food intake and could potentially represent one aspect of the CR pro-longevity mechanism.

Dr Mark Yeoman (School of Pharmacy and Biomolecular Sciences, University of Brighton, Brighton, UK) discussed the impact of gastrointestinal (GI) disorders on older adults and the problems associated with understanding the mechanisms involved. Fecal output is negatively affected by age and the percentage of compaction increases causing decreased colonic motility. These changes are suggested to be a result of reduced mucosal serotonin availability and a subsequent reduction in mucosal serotonin transporter (SERT) expression, which has been shown in aging [19]. Serotonin is the neurotransmitter responsible for nutrition absorption and motility in the GI tract. Found primarily in the enterochromaffin (EC) cells, serotonin allows communication between the gut lumen and neurons. Dr Yeoman suggested that aging is also associated with reduced amplitude and frequency of electrical signaling within colonic migrating motor complexes (CMMC), responsible for propelling fecal content along the large bowel. Additionally, he proposed that there is also an age-related loss of tachykinin NK_2_ mediated signaling in the GI with age, which would in turn affect the signaling of CMMC. Thus selective NK_2_ receptor antagonists can potentially reactivate inhibited motility in older adults.

Professor David Grundy (Department of Biomedical Science, University of Sheffield, Sheffield, UK) presented data examining GI sensory function of young and old mice. He showed that changes in the enteric nervous system with age results in increased incidence of constipation, rectal sensitivity and evacuation. This also appears to be related to dysfunction of the nervous system, as evident by older individuals with reduced rectal sensitivity and compliance. In this study, rectal distension caused increased jejunal mesenteric afferent discharge in both ages, but had reduced low threshold (LT) and high threshold (HT) neuron activity. Professor Grundy suggested that age attenuated all three subtypes of afferent neurons and reduced neuronal signaling, which may be a result of reduced serotonin signaling. 

Dr Sarah Rayment (Queens Medical Centre, University of Nottingham, Nottingham, UK) followed on by discussing the potential use of drugs not normally associated with fecal incontinence (FI). Currently, around 10% of community dwelling older adults and 50% of nursing home residents suffer from FI; however, very few therapeutic interventions are available. Dr Rayment’s previous work in sheep showed that α1-adrenoreceptor agonists, normally used for hypotensive patients, caused vasoconstriction in the sphincter and increased pressure in the bowel [20]. Furthermore, α2-adrenoreceptor agonists such as clonidine, normally used in hypertensive patients and for attention deficit-hyperactivity disorder (ADHD), appear to modify compliance and sensitivity of the sphincter.

Professor Alison Gurney (Faculty of Life Sciences, Manchester University, Manchester, UK) opened the second session on bowel dysfunction by discussing the role of potassium and calcium channels in smooth muscle function. Normal bladder contractions are dependent on increased Ca^2+^ release in the smooth muscle, and Ca^2+^ release can be generated in ‘sparks’ and ‘waves’ [21]. Smooth muscle contains K^+^ channels which set the resting membrane potential, and detect and respond to a rise in intracellular Ca^2+^. Loss of K^+^ channels results in bowel tissue dysfunction, for example mice which lack the large-conductance Ca^2+^ activated K^+^ channel (BK_Ca_) present with incontinence and an overactive bladder [22]. A lack of BK_Ca_ was shown to reduce spontaneous outward current even though Ca^2+^ concentrations in the bladder smooth muscle remained unchanged. With age there is an altered concentration of cyclic nucleotide phosphodiesterases (PDEs) and PDE-5 is known to relax muscle via a nitric oxide (NO) mechanism. Inhibition of PDE-5 increases cAMP and cGMP which act on BK_Ca_, allowing increased bladder control and relaxation. Furthermore, mice lacking BK_Ca_ and PKG1, activated upon binding with cGMP, do not have increased muscle relaxation [23]. Finally, drugs like sildenafil, which raise intracellular cGMP concentrations, promote BK_Ca_ activity and ultimately smooth bladder muscle relaxation, may have utility in older adults to improve continence.

Professor Christopher Fry (Institute of Biosciences and Medicine, University of Surrey, Surrey, UK) supplemented the earlier talk by Dr Rayment by discussing the sensory functions of the bladder in response to aging. An overactive bladder (OAB) is associated with increasing age, and can be characterized by symptoms of urgency, frequency and nocturia. OAB is a sensory and neural processing dysfunction rather than a motor disorder, since there is no age difference in bladder contractions. The normal function of the bladder involves release of the neurotransmitters acetylcholine (ACh), ATP and NO, which activate local afferent nerves and communicate with the central nervous system (CNS), or to perform functional contraction of the detrusor muscle. Furthermore, distension causes increased ATP release from the bladder epithelial cells and exerts its actions specifically through P2X_3_ ATP sensory receptor activation on the afferent nerve plexus. In older adults there is an increased release of ACh and ATP during distension-induced release leading to sensory dysfunction and OAB disorders [24]. Professor Fry suggested that further understanding of the sensory mechanisms attributed to OAB disorders will allow successful targets to be determined. Currently, therapeutic use of compounds such as oxybutynin, an antimuscarinic (ACh antagonist) used to treat intractable detrusor muscle overactivity, and vanilloids (capsaicin, resiniferatoxin) to attain bladder deafferentation, need further investigation to fully discern their actions.

Professor Khalil Bitar (Wake Forest Institute for Regenerative Medicine, Winston-Salem, NC, USA) ended the day with a fascinating description of how to potentially solve age-associated bowel and bladder dysfunction by bioengineering an internal anal sphincter (IAS). Within 5 days of culture, alignment of smooth muscle sheets was complete and the engineered IAS was both functional and produced force. Cells from enteric neurospheres were then co-cultured with IAS smooth cells using dual-layered hydrogels which permitted circular construct formation. This construct contracted in response to ACh and relaxed in response to cAMP, before implantation onto the back of an athymic (Rag1^−/−^) rat and neovascularized. The construct was morphologically good and responded to ACh stimulation. Following this the innervated human IAS construct was implanted *in situ* into the rodent. Neural networking and GI motility was restored using autologous cell sources and electrical field stimulation suggested functional capabilities [25]. Defecation was normal, further suggesting full functional capacity. These data represent the potential for overcoming GI problems in humans by replacing affected areas with bioengineered fully functional and intrinsically innervated human IAS tissue.

### New Dynamics of Ageing

The New Dynamics of Ageing (NDA) programme (http://www.newdynamics.group.shef.ac.uk) is a research initiative funded through a joint collaboration between five UK Research Councils. It supports multidisciplinary research to elucidate the forces driving the aging process and ultimately aims to develop methods and protocols to improve the quality of life of older adults. Three of the biogerontological researchers funded by the NDA presented their findings.

Dr Terence Davis (Institute of Cancer & Genetics, Cardiff University, Cardiff, UK) uses Werner syndrome (WS) fibroblasts to investigate cellular aging via modulation of the p38 stress-signaling pathway. Activity of this pathway has been shown to be augmented in WS forcing arrest of the cell cycle and subsequent senescence. Using p38 specific inhibitors, the team was able to investigate the exclusive action of p38 demonstrating a partial normalization of the growth of WS fibroblasts and confirming a role for p38 in the development of senescence [26]. However, *in vivo* efficacy and tolerance of these compounds are limited, with side effects including CNS inflammation and liver toxicity when tested in both mice and dogs. Consequently, the need for new targets and compounds with fewer side effects are required. Dr Davis and colleagues have been able to synthesize three new inhibitors specific for the major downstream target of p38, MK2. This is the major kinase responsible for phosphorylation of HSP27, the main driver behind F-actin stress-fiber formation which features prominently in young WS fibroblasts. Preliminary data suggest that inhibition of MK2 is able to marginally increase the growth potential of WS fibroblasts.

Dr Penelope Mason (Department of Biochemistry, University of Oxford, Oxford, UK) also uses WS as a model system and has created a plasmid containing miRNA targeted against the Werner protein (WRN), addition of which results in the reversible knockdown of WRN helicase and exonuclease in cells aged in culture [27]. The presence of WRN is thought to prevent premature aging by stabilizing the genome. Through utilization of this new system, Dr Mason aims to assess the role of DNA damage in the induction of senescence and its ability to drive the aging process. Additionally, she also presented work on the cellular alterations that take place during replicative senescence. Fibroblasts treated with rapamycin, an inhibitor of mammalian target of rapamycin complex 1 (mTORC1), exhibited a reduced growth rate and underwent a greater number of population doublings before becoming senescent. Dr Mason was able to show a significant down-regulation of proteins involved in the regulation of genome structure and stability upon inhibition of mTORC1. This illustrates a novel pathway in the development of replicative senescence.

To conclude the session, Niharika Arora Duggal (Medawar Centre for Healthy Ageing Research, University of Birmingham, Birmingham, UK) presented her work on the synergistic effects of emotional and physical stress on immunesenescence, and hypothesized that the synergistic effects of both stressors act to accelerate immunesenescence in older adults. Hip fracture is a common event in older adults and a major risk factor for progression to ill health and frailty. There are over 80,000 hip fractures each year in the UK alone with a 1-year mortality of 25%. In addition to this, one third of older adult patients with hip fractures go on to develop depression, which may further confound their ability to fully recover from the initial trauma. Ms Duggal demonstrated a reduction in both neutrophil superoxide production and natural killer (NK)-cell cytotoxicity in patients who suffered hip fracture and then went on to develop depression, whereas those with hip fracture alone did not show suppressed immunity. Thus treating depression in these patients may represent an effective route to improving outcomes after falls and hip fractures.

### Aging, regeneration and stem cells

Professor Valery Krizhanovsky (Weizmann Institute of Science, Rehovot, Israel) presented his work on the ability of senescent cells to limit tissue damage response. In the presence of an intact senescent machinery (INK4a, p53 and p21 pathways), the level of liver fibrosis is restricted; however, induction of senescence by knockdown of p53 and/or INK4a in hepatic stellate cells subsequently increases the levels of hepatic fibrosis. Using a model in which senescence has been disabled by knockdown of p53, Professor Krizhanovsky and colleagues demonstrated the ability of NK cells to recognize and subsequently clear senescent hepatic stellate cells. This is achieved through ligation of the NK cell receptor NKG2D with its ligands MICA and ULBP2 expression of which is up-regulated on the surface of senescent cells. The perforin and granzyme pathway then mediates killing of bound senescent cells, and their subsequent degradation and removal. Upon senescence, cells also up-regulate expression of decoy receptor 2 (DcR2), a molecule that inhibits the death receptor pathway by blocking TRAIL-R [28]. This protects senescent cells from NK cell killing mediated by the death receptor pathway, and instead restricts NK cell mediated killing to the perforin and granzyme pathway. Ultimately, a buildup of senescent cells with age may be the result of impaired immune surveillance and therefore decreased clearance of these cells.

Professor Janet M Lord (MRC-ARUK Centre for Musculoskeletal Ageing Research, University of Birmingham, Birmingham, UK) continued the immunity theme, and described her research of the decline of the innate [29] and adaptive immune systems. On the innate side, Professor Lord described how, with aging, NK cell numbers increase but their cytotoxic ability decreases. This decrease is due to reduced perforin release at the immune synapse, the contact point between the NK cell and its target, due to a lack of localization of perforin secretory granules to the synapse [30]. Together with Professor Krizhanovsky's findings, these data may help to explain why senescent cells accumulate with age. She also discussed the possible causes of the age-related increase in inflammatory cytokines, so-called inflammaging, [31] how lifestyle factors are key drivers of this phenomenon and latent cytomegalovirus (CMV) infection can be ruled out as a causative agent [32].

Although the negative effects of advancing age on T cell development have been widely reported in literature, it remains unclear as to whether these effects are due to changes in the thymic microenvironment, a reduction in the number of stem cells feeding the thymus or the presence of intrinsic defects within the stem cell compartment itself. To address this, Professor Richard Aspinall (Translational Medicine, Cranfield University, Cranfield, UK) developed a model system using both human keratinocyte and fibroblast cell lines grown on a synthetic matrix, which allow the deconstruction of the thymic microenvironment. By seeding CD34^+^ stem cells isolated from cord blood onto the meshed thymus, he was able to demonstrate the development of immature (CD4^+^ or CD8^+^) and mature (CD4^+^ or CD8^+^) thymocytes. However, when stem cells from old blood were seeded into the meshed thymus, there was a distinct lack of T cell development occurring suggesting an intrinsic defect in the stem cells isolated from old blood.

Dr Helen A Thomason (Manchester Immunology Group, University of Manchester, Manchester, UK) presented her work on the role of protein kinase C alpha (PKCα) in the regulation of desmosomes and wound healing [33]. Desmosomes are adhesion complexes found in the cell membrane, which mediate cell-to-cell adhesion and maintain the integrity of the epithelium. Upon wounding these complexes switch from a Ca^2+^-independent ‘super-adhesive’ state to a Ca^2+^-dependent non-adhesive state, allowing keratinocyte migration and subsequent re-epithelialization. Older adults have an increased susceptibility to development of chronic wounds with 5% developing leg ulcers which, if left untreated, can become chronic. Dr Thomason and colleagues showed that expression of genes encoding desmosomal proteins was decreased in older adults with desmosomes remaining in a Ca^2+^-independent, ‘hyper-adhesive’ state. This prevents keratinocyte migration and results in delayed wound-healing. PKCα regulates the switch between the Ca^2+^-independent and Ca^2+^-dependent states, and in chronic, non-healing wounds of older adults, PKCα failed to co-localize with the membrane bound desmosome preventing the switch to a Ca^2+^-dependent state, thus preventing wound healing. A mouse model expressing a constitutively active form of PKCα showed an increased rate of re-epithelialization and faster wound healing.

To conclude the session, Song Baik (School of Immunity and Infection, University of Birmingham, Birmingham, UK) presented her work on the reversal of thymic atrophy. Using inducible pluripotent stem (iPS) cells produced from somatic cells and re-programming them back to pluripotency, she was able to produce a fully functioning thymus *in vivo*. By injecting iPS cells into a blastocyst of a nude mouse, Ms Baik and colleagues were able to ‘grow’ a thymus, with the correct thymic architecture in an otherwise athymic mouse. In addition, the iPS thymus was fully functional containing all four T cell developmental subsets, and produced T cells that were able to migrate into secondary lymphoid tissue and respond to activation. The potential of iPS cells to be used to combat thymic atrophy in older adults by regenerating an older thymus is therefore an interesting and realistic concept.

### Pathways, molecules and interventions

The final session of the meeting considered ways to intervene in improving lifespan. Professor Gordon Lithgow (Buck Institute for Research on Aging, Novato, CA, USA) presented a role for genes and small molecules in enhancing lifespan, as well as being potential pharmaceutical targets. Professor Lithgow suggested that maintenance of protein homeostasis was a key mediator in lifespan extension. Increased expression of protein chaperones and heat shock proteins are associated with longevity, whereas aging is associated with a loss of protein homeostasis and an accumulation of insoluble protein aggregates. Professor Lithgow chose the relationship between normal aging and neurodegenerative disorders to assert his point. Neurodegenerative pathologies such as Alzheimer’s disease and Parkinson’s disease have an accumulation of insoluble protein aggregates which contribute to disease severity. Normal aging also involves increased production of protein aggregates and he demonstrated that reduction of aggregates in nematodes significantly increases lifespan [34]. Screening for small molecules in the Lithgow Lab, which may retard the aging process, revealed some interesting candidates: exposure to Thioflavin T (ThT), curcumin and rifampicin block early protein aggregation, reduce pathology and increase lifespan in *C Elegans*[35]. The mechanism of ThT actions appear to be dependent on a protein homeostasis network regulated by heat shock factor 1 (HSF-1), transcription factor SKN-1 and autophagy. Finally, Professor Lithgow described the role of metal homeostasis (metallostasis), and the link between increased metals such as aluminum and neurologic disease and protein aggregation. He suggested that aluminum accumulation alters the composition of other metals and reduces lifespan [36].

Investigating a second mechanism for lifespan extension, Dr Colin Selman (School of Biological Sciences, University of Aberdeen, Aberdeen, UK) presented work on insulin/IGF-1 signaling (IIS) and its effects on cellular stress resistance. Reductions in IIS increase lifespan via reduction in insulin receptor substrate 1 (IRS1) levels [37]. Previous work has demonstrated that this lifespan extension is not due to alterations in anti-oxidant protection, reduced oxidative damage or enhanced base excision repair. Examination of the effects of oxidative and non-oxidative stressors via exposure to hydrogen peroxide, cadmium and methyl methanesulfonate on the survival of fibroblasts isolated from IRS1 null mice revealed no differences. Instead Dr Selman agreed with the proposals of Professor Lithgow, that there was a link between cellular stress resistance and protein aggregation. In particular, he suggested a role for endoplasmic reticulum stress in disrupting protein folding homeostasis, contributing to age-related pathology and that the IRS1^−/−^ mice were more resistant to this stress.

Disuse atrophy is commonly seen in the older adult population as a result of age-related inactivity. In a number of conditions, angiotensin II type I receptor blocker (ARB), losartan, has produced beneficial effects in ameliorating muscle atrophy. However, these effects seem to be age-dependent, as shown by Tyesha Burks (School of Medicine, Johns Hopkins University, Baltimore, MD, USA). In young mice, losartan was unable to protect from disuse atrophy and only conferred partial protection in adult mice, but was able to completely protect against the loss of muscle mass in older mice [38]. In older mice, losartan prevents angiotensin II from binding to angiotensin receptor 1 (ART1), thereby modulating activity of the transforming growth factor-β signaling pathway known to be downstream of ART1. In addition, losartan appears to be able to modulate the Akt/mTOR/FOXO3A pathway in an age-dependent manner, a pathway already known to be involved in the loss of muscle mass due to physical inactivity.

To conclude the annual scientific meeting, Professor Richard Faragher (School of Pharmacy and Bimolecular Sciences, University of Brighton, Brighton, UK) gave a short talk on the relationship between resveratrol and replicative senescence. Aging can be modulated by environmental factors including CR and single gene mutations such as *daf2*, *Chico* and *Igfr1*. The promise of aging research is to mimic these environmental factors and the use of resveratrol has been widely proposed as a CR mimetic by acting through sirtuin1 (SIRT1). However, its precise mode of action in relation to lifespan extension remains unclear, although its anti-cancer activity is thought to contribute. Professor Faragher and colleagues reported that exposure of fibroblasts to resveratrol induces reduced proliferation and p38 MAPK-dependent senescence *in vitro*. However, circulating levels of resveratrol achieved in supplementation studies remain too low to be able to induce replicative senescence. Professor Faragher concluded that resveratrol altering cellular turnover as the mechanism of anti-aging effects is unfounded [39].

## Abbreviations

ACh: acetylcholine; ARB: angiotensin II type I receptor blocker; ART1: angiotensin receptor 1; AVP: arginine vasopressin; BBSRC: Biotechnology and Biological Sciences Research Council; BSRA: British Society for Research on Ageing; CGMP: cyclic guanosine monophosphate; CMMCs: colonic migrating motor complexes; CMV: cytomegalovirus; CNS: central nervous system; CR: caloric restriction; DcR2: decoy receptor 2; EC: enterochromaffin; FI: fecal incontinence; FIRKO: fat-specific insulin receptor knockout; GC: glucocorticoids; GH: growth hormone; GI: gastrointestinal; HPA: hypothalamic-pituitary-adrenal; HSF-1: heat shock factor 1; HT: high threshold; IAS: internal anal sphincter; ICC: interstitial cells of Cajal; IGF: insulin-like growth factor; IIS: insulin/IGF-1 signaling; IPS: inducible pluripotent stem; IRS1: insulin receptor substrate 1; LT: low threshold; miRNA: microRNA; mTOR: mammalian target of rapamycin; mTORC1: mammalian target of rapamycin complex 1; NK: natural killer; OAB: overactive bladder; PDE: phosphodiesterase; PKCα: protein kinase C alpha; PKG: protein kinase G; SERT: serotonin transporter; ThT: Thioflavin; TRAIL-R: tumor necrosis factor-related apoptosis-inducing ligand receptor; WRN: Werner protein; WS: Werner syndrome

## Competing interests

The authors declare that they have no competing interests.

## Authors’ contributions

HG and DBB contributed equally to the manuscript preparation and should be considered as joint first authors. Both authors read and approved the final manuscript.
